# Rare case of low-grade fibromyxoid sarcoma of the thoracic wall with complete sternum reconstruction

**DOI:** 10.1080/23320885.2022.2064290

**Published:** 2022-05-13

**Authors:** João Nunes Pombo, Artur Nixon Martins, Catarina Paias Gouveia, Ágata Nawojowska, Samuel Mendes, Daniel Cabral, Francisco Félix, Bruno Rosa, Carlos Pinheiro, Miguel Andrade, Gaizka Saenz Ribeiro

**Affiliations:** a Serviço de Cirurgia Plástica, CHULN – Hospital de Santa Maria, Lisbon, Portugal; b Serviço de Cirurgia Torácica, CHULN – Hospital Pulido Valente, Lisbon, Portugal

**Keywords:** LGFMS, low-grade fibromyxoid sarcoma, sternal reconstruction

## Abstract

An 18-year-old boy presented with a giant midline mass with 9 years of evolution. The tumor was excised, and reconstruction made with a customized sternum implant and a free latissimus dorsi muscle flap with skin graft. Histological analysis was compatible with low-grade fibromyxoid sarcoma (LGFMS).

## Introduction

Low grade fibromyxoid sarcoma (LGFMS) was first described by Evans in 1987 [1].

LGFMS is an extremely rare malignant tumor that appears in young adults, with a reported incidence of 0.18 per million [2,3]. It consists of bland appearing spindle cells in a collagenous and myxoid matrix [1–6].

LGFMS usually presents as a painless, slow-growing mass, within the deep tissues of the extremities and trunk, but rarely arising primarily from the chest wall. It has been described in other unusual locations such as the head and neck or abdominal cavity. Metastasis can be present, usually late in the disease, mainly to the lung [2–5].

Herein we report a case of a giant thoracic LGFMS with origin on the sternum.

## Case report

**Figure 1.**
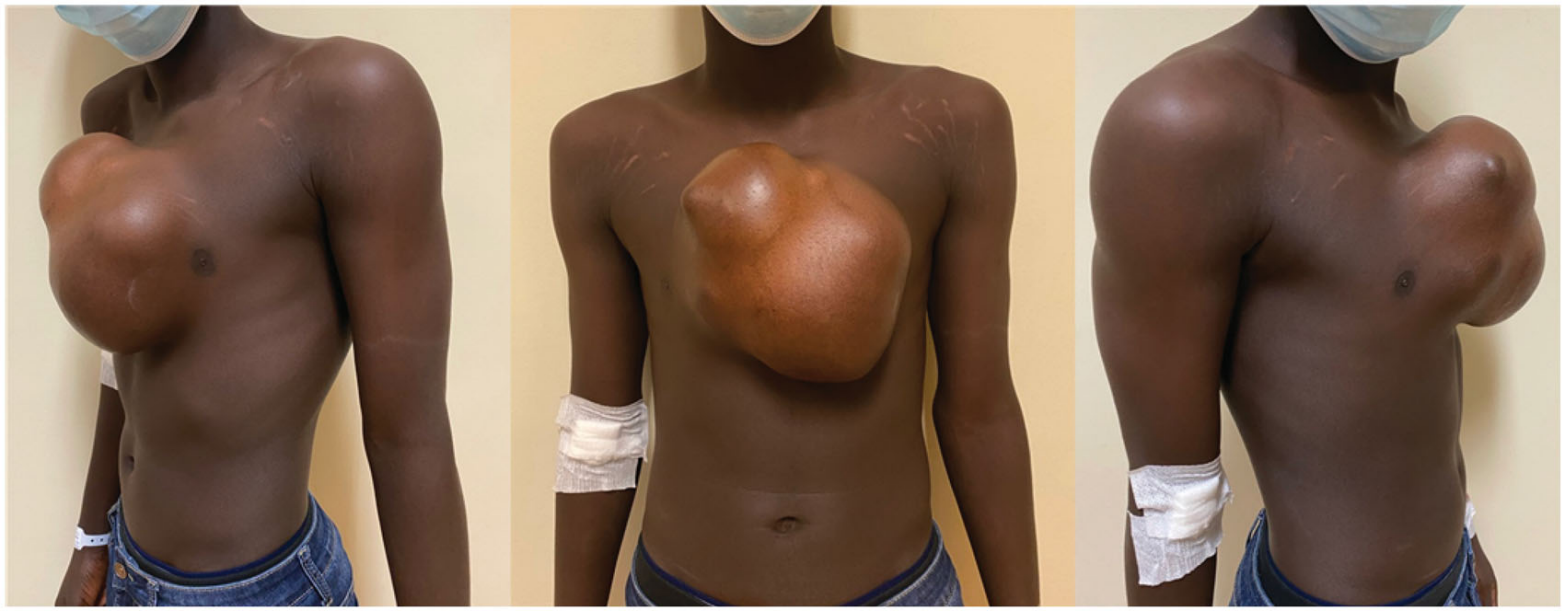
Pre-operative photographs of the patient.

An 18-year-old boy was evacuated from his home country (Guinea-Bissau) to Portugal because of a giant midline thoracic mass, that had been slowly growing for the last 9 years (Figure 1). The patient had no relevant medical history. He presented no pain or other complaints beside the obvious aesthetic deformity and limitation of physical activity.

**Figure 2.**
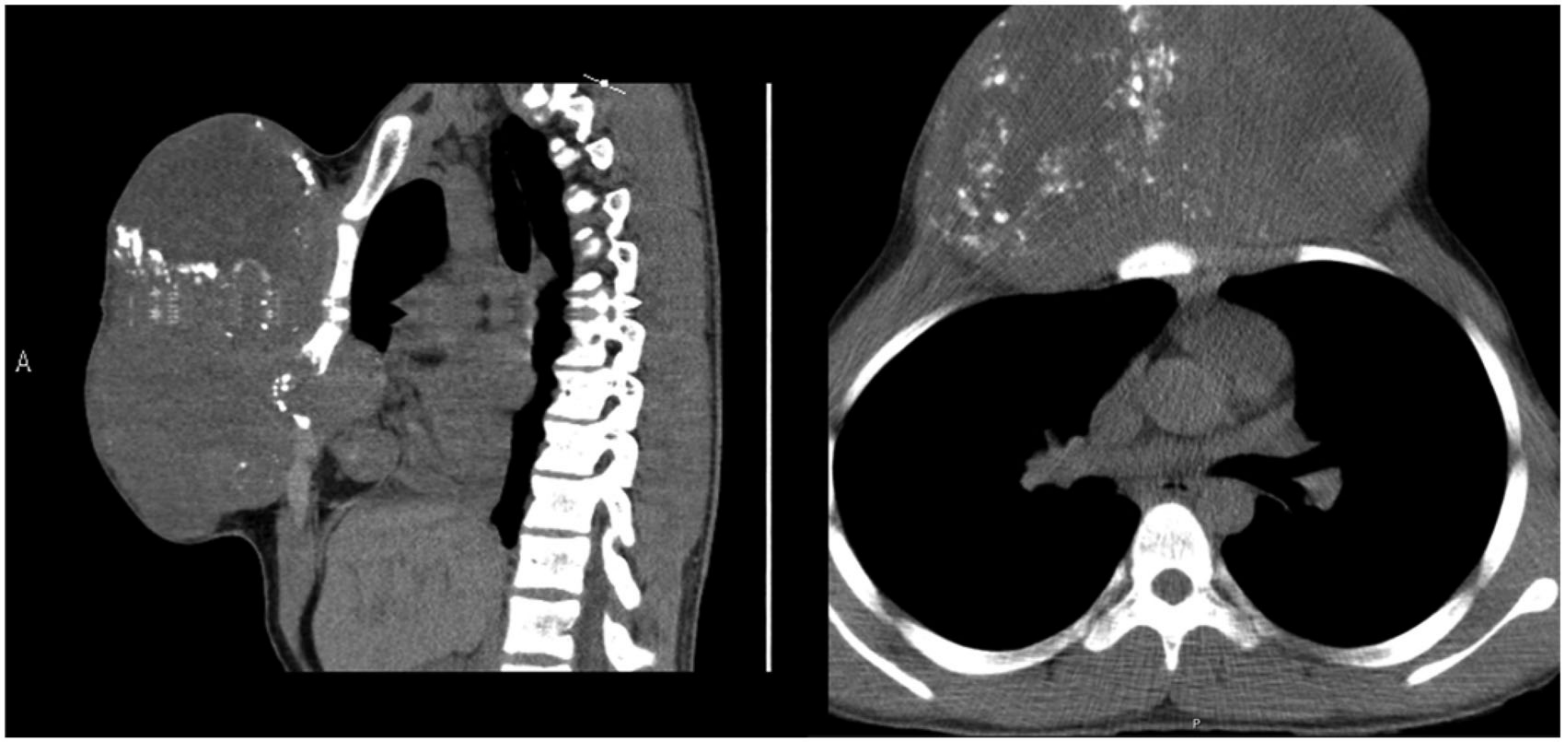
Preoperative CT scans.

A CT scan was made which identified a 17 × 17 × 12 cm heterogeneous mass with signs of disperse calcification, arising from the body of the sternum (Figure 2). Several biopsies’ attempts were made but results came inconclusive.

A combined Thoracic Surgery and Plastic Surgery approach was planned, and removal of the complete sternum was agreed upon. Therefore, a custom-made thoracic implant was designed based on the CT scan.

**Figure 3.**
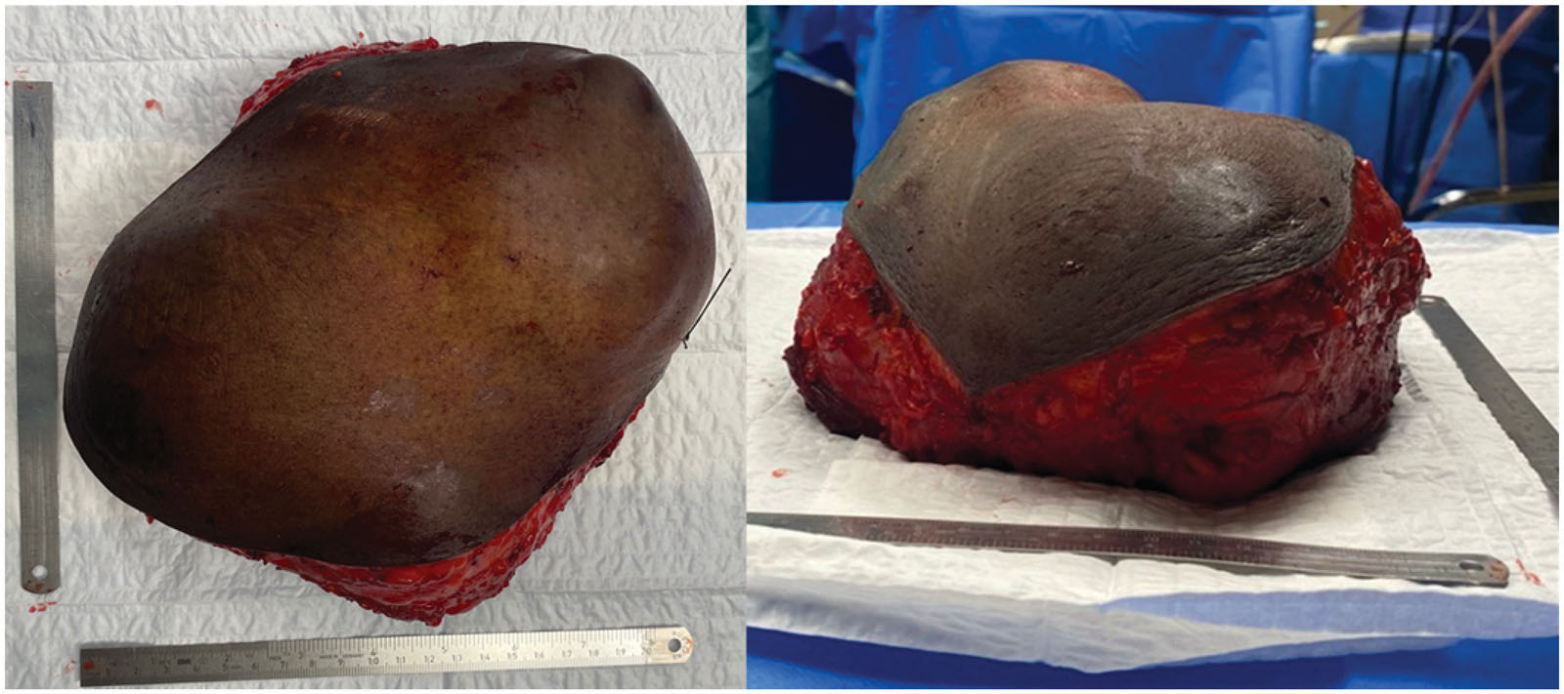
Tumor after resection. Notice the base of the tumor to be slightly smaller than the superficial component.

**Figure 4.**
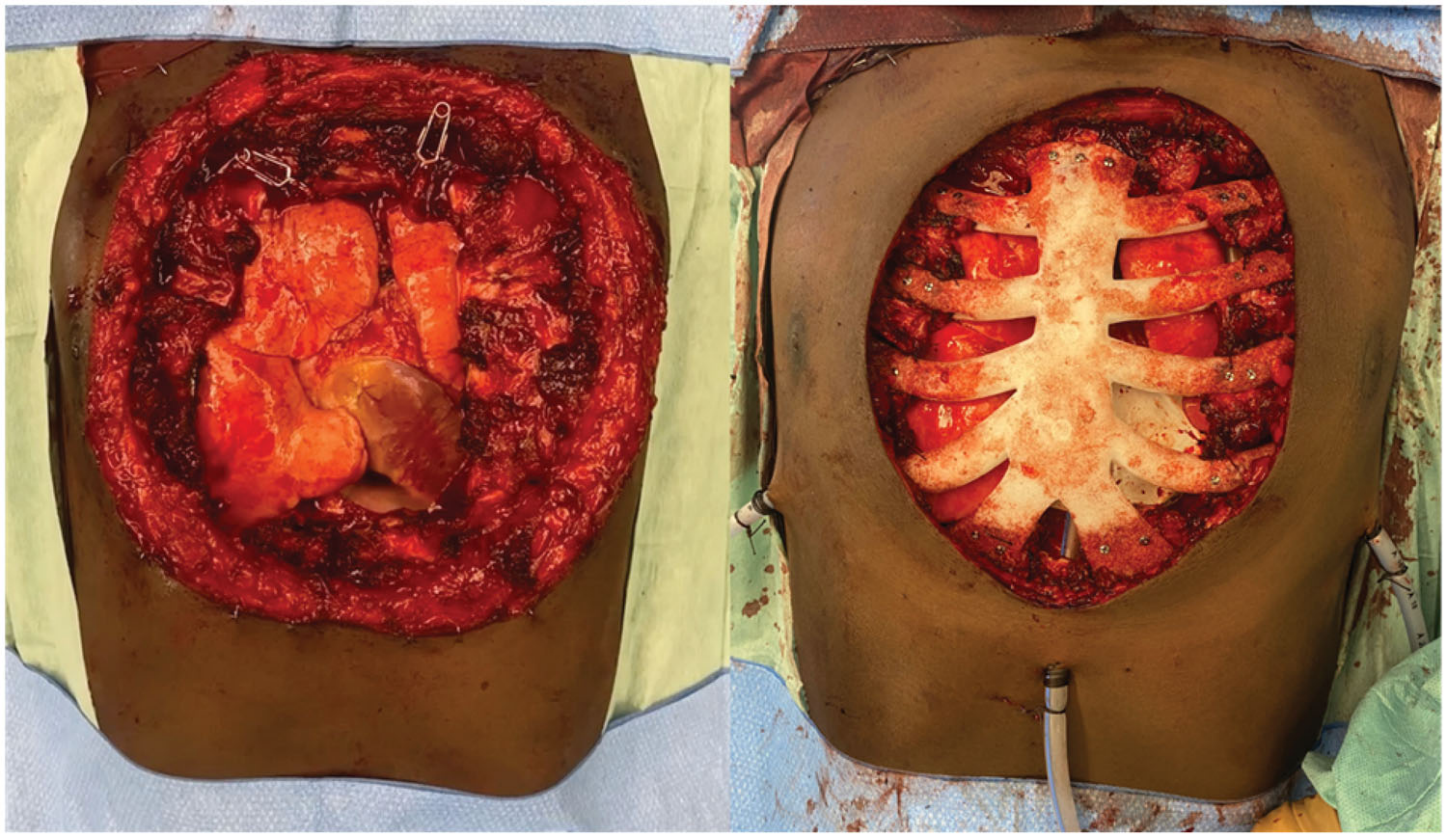
Intraoperative views after tumor removal (left) and after sternal reconstruction with a *StarPore*™ custom-made prostheses.

**Figure 5.**
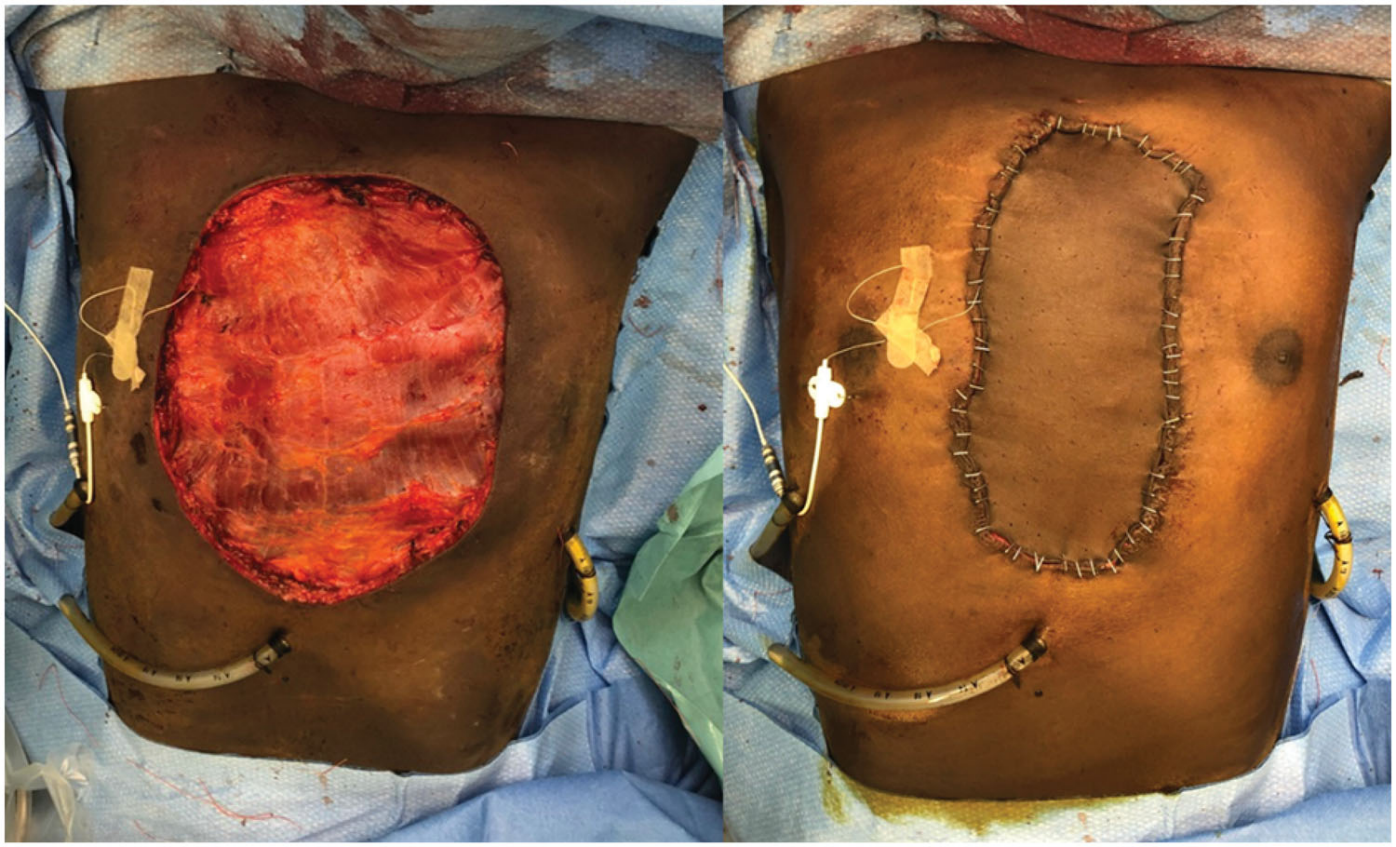
Intraoperative view with LD muscular free flap (left) and skin graft (right).

For the surgery, patient was placed supine and the mass was excised respecting 2 cm of macroscopic margins, with intra-operative finding of pericardial invasion by the tumor. Final defect was 20 × 20 cm. After surgical excision, a pericardial mesh was used to reconstruct the pericardium and the sternum prosthesis fixed to the remaining ribs (Figures 3 and 4). Intrathoracic drains were used. A steri-drape™ was used to cover the wound and the patient placed on lateral decubitus to raise a free latissimus dorsi muscle flap. The flap was inset to protect the entire thoracic prosthesis and the anastomosis made to the right internal mammary vessels (Figure 5). A partial thickness graft was used to cover the muscle (Figure 5). Postoperative course was uneventful.

Histological and immunohistochemical analysis revealed LGFMS with free margins, and exclusive and multifocal MUC4 expression, respectively.

**Figure 6.**
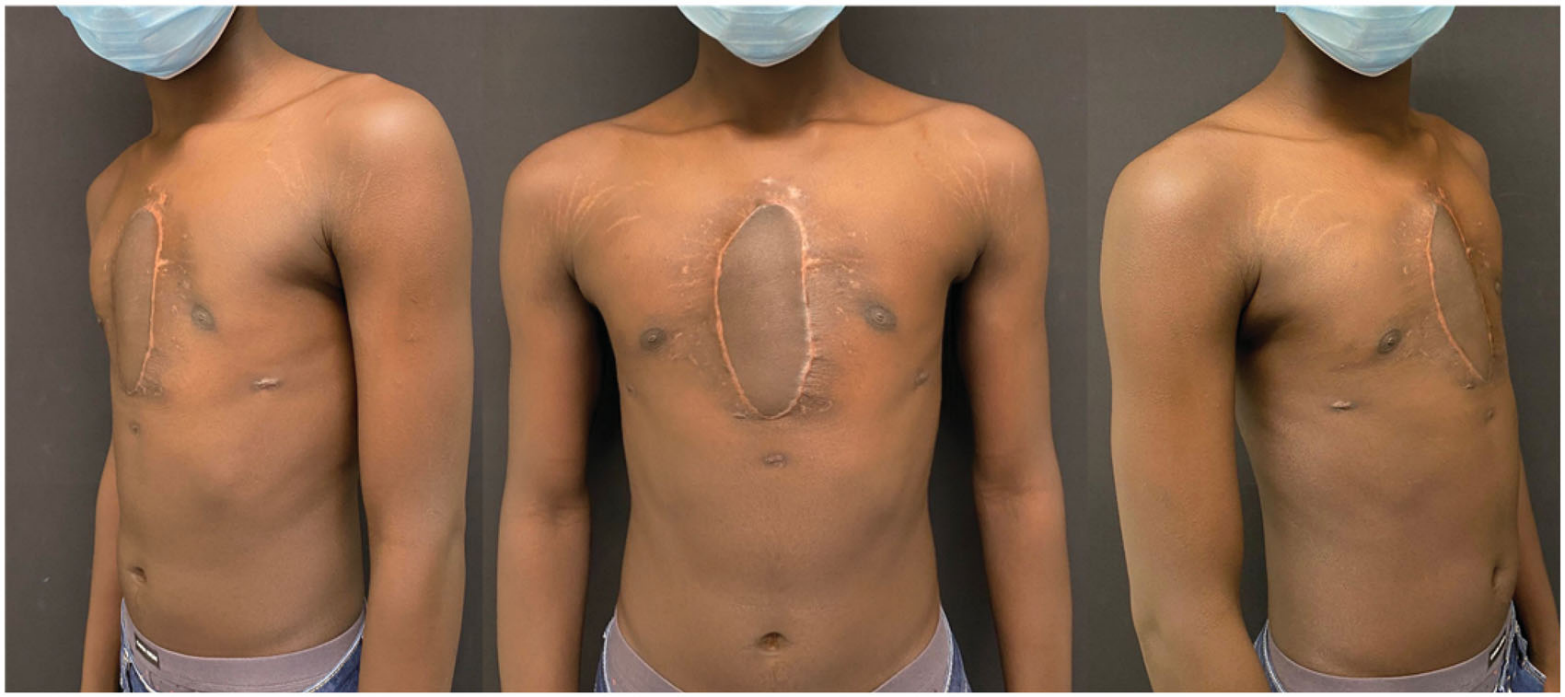
Post-operative at 6 months.

At 6 months of follow-up the patient presented no signs of recurrence and has no physical activity restraints (Figure 6).

## Discussion

The majority of chest wall tumors are malignant and arise from metastasis or direct invasion from adjacent tumors of the thorax, mediastinum or soft tissue, more frequently from breast and lung cancers. Primary chest wall tumors are relatively uncommon [7]. Around half of them are from soft tissue origin [8]. Benign tumors include osteochondroma, chondroma, fibrous dysplasia, eosinophilic granuloma, and giant cell tumor. Malignant entities are comprised by osteosarcoma, chondrosarcoma, solitary plasmacytoma, Ewing sarcoma and soft tissue sarcomas [8].

LGFMS is a malignant tumor which often misleads pathologists for its innocuous histological appearance. It was first described by Evans and has been since then accepted as an individual entity.

The tumor is more common in young adults (median age of 32.5 years), even though it can affect all ages and almost one fifth of the cases may present in the pediatric age [2,3]. There appears to be a male predominance, especially in the younger population. It usually presents as painless, deeply located, slow-growing mass, in the extremities, more commonly the lower extremities, or trunk. Patients can report a growing mass for months or even years before the diagnosis. Other areas have been reported such as head and neck, mesentery, omentum, kidney, heart, or the anal sphincter [2–6,9].

Our case appears to have origin in sternum-chondral junction with posterior growth into the thoracic cavity. Other reports of chest wall LGFMS have been reported with a variable clinical spectrum – from asymptomatic growing tumor to persistent cough or acute respiratory distress [3,5,9–14]. Sajid et al. reviewed the reported cases of LGFMS with mediastinal occurrence and identified 7 other cases, totaling 8. The author concluded mediastinal tumors arise in the same population and do not seem to present a different clinical behavior [3].

Pediatric patients usually present with more superficial forms of LGFMS and appear to have better prognosis. Small size (<3.5 cm) also seems to a good prognosis factor [14].

Histologically, LGFMS is characterized by bland appearing spindle cells in a patternless or whorled growth pattern in a combination of myxoid to highly collagenous/fibrous stroma. Mitotic figures are absent or rare, and cellularity is typically low even though myxoid areas present more cellularity compared to the fibrous ones. The former also present a more developed vascular network [15]. Pericollagenous rosettes, hypercellularity and other variable features have been described, but the differences do not seem to affect tumor behavior or overall survival [2,4]. Immunohistochemically, the tumor is typically positive for MUC4 and negative for CD-34, S-100 protein, ALK-1 SMA [2–6,12,14]. Cytogenetic analysis shows aberrancies with t(7;16) (q34;p11) translocations resulting in fusion of *FUS* and CREB3L2 genes, with its resulting chimeric protein present in 95% of well-defined LGFMS [2–6,9,12–14].

Treatment consists of surgical resection with clear margins. Chemotherapy and radiotherapy have been used, particularly in positive resection margins, but its efficacy is still up to debate [2,3]. One of the major issues when addressing these tumors is complete resection as many tumors by the time of surgery have grown to sizes as big as 20 cm, especially if invading the chest wall. Partial anterior and posterior chest wall resections have been described in the literature but might present with incomplete margins [5,9–11]. We opted for total sternal and sternal-chondral junctions resections to be able to get adequate margins and a better clinical outcome.

Sternal reconstruction is essential for protection of the mediastinal contents, stabilization of the thorax and for maintenance of respiratory physiology. Usually, titanium plates or meshes are the chosen method for reconstruction but molding of the plates is dependent on surgeon’s experience. Recently, there has been an interest in developing 3 D printed custom-made prostheses for total sternum reconstruction [16]. These prostheses have evolved from titanium to high-density porous polyethylene (*StarPore*™ – *Anatomics*™), which is significantly lighter and more flexible. It also allows intraoperative modification and fast tissue integration [15]. We have selected a *StarPore*™ prostheses, manufactured from a preoperative CT of the patient. For soft tissue reconstruction a latissimus dorsi muscle free flap was chosen as it presented adequate size and thickness to fully cover the prosthesis and the soft tissue defect.

Recurrences and metastases have been described in the literature, usually ranging from 9 to 21% and 6 to 27%, respectively [4,17]. However, Evans with a follow-up time of 14 years described in a series of 12 cases a local recurrence rate of 68% and a 41% rate of metastases [6]. It seems these numbers might be exaggerated as more recent reports show a less aggressive behavior. LGFMS metastasizes frequently late in the disease, mainly to the lung, with Guillou reporting a median time to metastasis of more than 9 years in 83% of the metastatic cases [17]. This remains a major limitation for most studies as long follow-ups are not available for most series. Metastatic probability appears to be related to the mass growing time. Thus long-term follow-up should be considered in these patients, even if free margins are achieved. In Sajid et al. series of mediastinal LGFMS, two recurrences were diagnosed after 7 and 9 years, reinforcing the need for long follow-up [3].

## Conclusion

We present a rare case of LGFMS in an unusual location. These tumors should be considered in the differential diagnosis of deep-seated masses of the thoracic wall, especially if long-term growth is noted. Correct diagnosis is of particular importance as long follow-up must be considered is these patients.
